# Additional Progress in the Development and Application of a Direct, Rapid Immunohistochemical Test for Rabies Diagnosis

**DOI:** 10.3390/vetsci5020059

**Published:** 2018-06-20

**Authors:** Charles E. Rupprecht, Zhiquan Xiang, Alexandre Servat, Richard Franka, Jordona Kirby, Hildegund C. J. Ertl

**Affiliations:** 1 LYSSA LLC, Atlanta, GA 30333, USA; 2 The Wistar Institute, Philadelphia, PA 19104, USA; jxiang@wistar.org (Z.X.); ertl@wistar.org (H.C.J.E.); 3 OIE/WHO/EU Laboratory for Rabies and Wildlife, French Agency for Food, Environmental and Occupational Health and Safety, 54220 Malzeville, France; Alexandre.SERVAT@anses.fr; 4 CDC, Atlanta, GA 30333, USA; rpf5@cdc.gov; 5 USDA, APHIS, Wildlife Services, Milton, FL 32583, USA; jordona.d.kirby@aphis.usda.gov

**Keywords:** diagnosis, direct rapid immunohistochemical test, fluorescent antibody test, lyssavirus, proficiency testing, rabies, sensitivity, specificity, surveillance, zoonosis

## Abstract

Laboratory-based surveillance is fundamental to effective rabies prevention and control. The direct fluorescent antibody (AB) test (FAT) is the gold standard for rabies diagnosis. Recently, additional tests besides the FAT have been developed, such as the direct rapid immunohistochemical test (DRIT). In this study, our objective was to further refine technical aspects of the DRIT using a combination of two monoclonal ABs (MABs), 502 and 802, conduct additional testing among rabies reference laboratories using a diversity of animal species and rabies virus (RV) variants and compare the potential utility of the DRIT for end users via proficiency testing (PT) against the FAT. Considering the ideal molar ratios of biotin to AB in formulation of the DRIT conjugate, 3.9 was found to be superior to 7.4, for detection of RV antigens in the brain of a naturally infected raccoon. Optimization of the DRIT conjugate may also be dependent upon the apparent choice of specific viral antigens for testing, as a gray fox RV variant reacted less strongly than a raccoon RV variant in determining the working dilution of the MAB cocktail. Using the same MABs and protocol, the DRIT was compared to the FAT using more than 800 samples of mammalian brains, representative of more than 25 taxa, including in excess of 250 animal rabies cases from Europe and North America. Sensitivity was determined at 98% (96–100%, 95% CI) and specificity was calculated at 95% (92–96%, 95% CI). In a comparison among end users, PT of laboratory personnel resulted in values of 77–100% sensitivity and 86-100% specificity. Based upon these and previously reported results, the DRIT appears to be a suitable alternative to the FAT for use in lyssavirus diagnosis.

## 1. Introduction

Rabies is an acute progressive encephalitis caused by negative-stranded RNA viruses in the family Rhabdoviridae, genus *Lyssavirus* and a major neglected zoonotic disease with substantial agricultural and public health burden [1]. The current gold standard for rabies diagnosis is the direct fluorescent antibody test (FAT), which detects viral antigens in the brain of affected mammals [2]. While the FAT is highly sensitive and specific, this test requires the use of a fluorescence microscope, which may limit its application in some resource-poor countries. In support of a global plan for the elimination of canine rabies and for ongoing regional wildlife vaccination programs, additional diagnostic tests are needed [3].

The direct rapid immunohistochemistry test (DRIT) was developed in the late 1990s, as an alternative to the FAT, for confirmatory diagnostic testing and to enhance laboratory-based surveillance of rabies in a de-centralized manner [4]. As in the FAT, the DRIT detects rabies virus (RV) antigens within brain impressions obtained from potentially rabid mammals. In contrast to the FAT, the DRIT uses formalin as a fixative. It furthermore uses anti-RV nucleoprotein antibodies (ABs), either monoclonal (M) or polyclonal (P) conjugated to biotin, a streptavidin-peroxidase enzyme and a chromogen reporter, such as acetyl 3-amino-9-ethylcarbazole (AEC). A light microscope can detect viral inclusions within infected tissues. Presently, with the exception of the anti-RV MABs or PABs (which may be self-produced or obtained from the OIE/WHO rabies reference laboratories), all of the other test reagents for the DRIT are available commercially (e.g., distilled water, PBS, TWEEN, formalin, etc.). After a series of incubations, washes and staining, DRIT results are available in ~1 h. Since its original development, the DRIT has been used in Africa, Asia, Europe, the Middle East and the Americas [4,5,6,7,8,9,10,11,12,13,14,15]. Using either MAB or PAB preparations, preliminary sensitivity and specificity values were deemed comparable to the gold standard FAT, with the majority of studies demonstrating complete test agreement, especially when fresh brain samples were tested [4]. 

The utility of the DRIT for consideration as a routine diagnostic assay is to estimate the prevalence of RV infection during enhanced surveillance and to facilitate risk analysis and implementation of prevention and control measures as regards to the spatio-temporal distribution of disease, such as during pathogen discovery, oral wildlife vaccination or elimination of canine rabies by mass immunization programs. As the DRIT becomes more widely used under different surveillance settings, several additional technical aspects should be analyzed, so that the protocol can be further optimized. The objectives of this study were to: compare the potential effect of biotin concentration on conjugate performance; investigate the possible influence of different antigenic RV variants upon the selected AB working dilution; provide comparative testing data using the same protocol and MABs to assess various parameters of the DRIT for basic rabies diagnosis using a diverse array of suspect animal brain samples; and to review proficiency testing (PT) results among end users.

## 2. Materials and Methods

### 2.1. Samples

The brainstem from a diversity of rabid and non-rabid animals was selected as the primary CNS tissue of choice. Over 800 individual samples, representing more than 25 Old and New World domestic animal or wildlife taxa, were compared by the DRIT and FAT (Table 1). Specimens originated from archived, road-killed or live-trapped and euthanized potentially rabid animals, collected as part of routine reference laboratory performance, surveillance activities or outbreak investigations during 2015–2016, in Europe and North America.

### 2.2. Antibodies

The mouse anti-RV nucleocapsid MABs 502 and 802 were selected as primary detection ABs, due to expected pan-reactivity or performance in viral antigen detection within formalin fixed CNS tissues, as previously described [16,17]. As the degree of biotin conjugation may impact AB performance, the effect of the relative amount of biotin (NHS-LC-Biotin, Thermo Scientific, Waltham, MA, USA) conjugated to MAB 502 was compared at 7.4 vs. 3.4 moles/mole protein for optimal test performance, using serial dilutions of the conjugated MAB (1.2 mg/mL, initial concentration) in PBS to detect the distribution, abundance and appearance of RV inclusions in rabid raccoon (*Procyon lotor*) brain impressions, according to the DRIT protocol.

### 2.3. Rabies Virus Variant Comparison

Several different RV variants may perpetuate among animal reservoir populations within surveillance regions [1,3,4,17,18]. To determine if the RV antigenic variant may have an influence on DRIT performance, aliquots of frozen CNS tissues of a naturally infected raccoon (*P. lotor*) from the eastern USA and a naturally infected gray fox (*Urocyon cineroargenteus*) from Texas, USA, were selected for study, representative of their importance within North America as major reservoirs and focus for oral vaccination efforts [5,18,19]. The effective working dilution of the biotin-conjugated 502 MAB for optimal detection of RV inclusions was compared within the CNS tissues of these RV-infected animals, representative of distinct antigenic and genetic origins [17,18]. 

### 2.4. Protocol

The direct FAT and basic DRIT were performed as described [2,20,21]. Briefly for the DRIT, glass microscope slides with animal brain impressions were air-dried, fixed in 10% buffered formalin for 10 min, dip-rinsed in PBS containing 1% Tween-80, immersed in 3% hydrogen peroxide for 10 min, and dip-rinsed in fresh PBS-1% Tween-80. Excess liquid was removed after each rinse by blotting the edges surrounding the impressions. The slides were incubated at ambient temperature with the biotinylated mouse anti-RV MABs for 10 min, dip-rinsed in PBS-1% Tween-80, incubated with streptavidin-peroxidase for 10 min and dip-rinsed in PBS-1% Tween-80. The selected chromogen substrate was prepared by adding 1 mL of AEC to 14 mL of 0.1 mol/L sodium acetate (pH 5.5), and 0.075 mL of 3% hydrogen peroxide. The slides were incubated with the AEC-peroxidase substrate for 10 min and dip-rinsed in distilled water. Slides were counterstained with 1:2 Gill’s hematoxylin for 2 min and dip-rinsed in distilled water. The stained impressions were mounted with a water-soluble mounting medium and examined by light microscopy for typical RV inclusions.

### 2.5. Overall Test Performance

Combining the data obtained from FAT and DRIT testing of the 816 animal samples in Table 1, diagnostic parameters, including sensitivity and specificity, as well as positive and negative predictive values, were determined.

### 2.6. U.S. National Inter-Laboratory Testing of Reference Brain Samples as A Surrogate for Reproducibility

In the USA, each year approximately 100,000 animals are examined for RV infection [18]. Laboratories conducting rabies diagnosis enroll in PT, typically for the FAT, but also for the DRIT. During 2015–2017, up to 16 different USDA, Wildlife Services facilities and 1 state laboratory participated in national DRIT PT, as offered nationally by the Wisconsin State Laboratory of Hygiene (http://www.slh.wisc.edu/proficiency/). All USDA, Wildlife Services field laboratories used the same DRIT protocol and the same lot of MABs. The PT samples included brain impressions on Teflon-coated microscope slides from wildlife or domestic mammals from the USA, which were suspected of RV infection. The PT was used for confirmation of the presence or absence of the pathogen, as previously determined by the FAT for detection of viral antigens in the brain of the potentially rabid mammal. The geographical locations of the animals varied, but all samples occurred in known rabies enzootic areas/regions. The actual date of sample collection varied by season during 2015–2017. A single result was due from each laboratory by a specified time, without the ability for re-testing. Wherever possible, additional information was included *ad hoc*, after the PT results were provided. Results were compiled, once the PT was finalized.

## 3. Results

**Figure 1:**
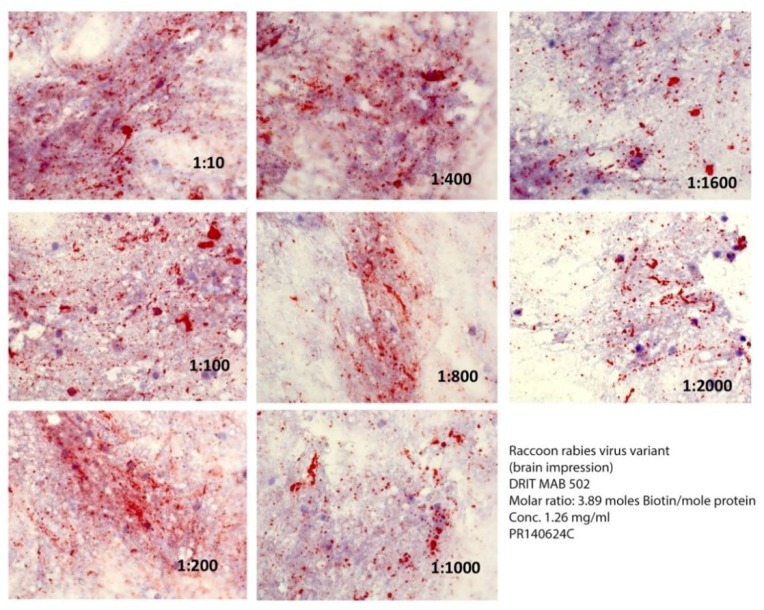
Comparative reactivity of serial dilutions of the DRIT MAB 502 (in PBS) at a ratio of 7.4 moles of biotin per mole of protein against a raccoon rabies virus variant. Rabies virus antigens appear as magenta inclusions against the bluish-purple background of uninfected CNS tissue in Figure 1, Figure 2 and Figure 3.

**Figure 2:**
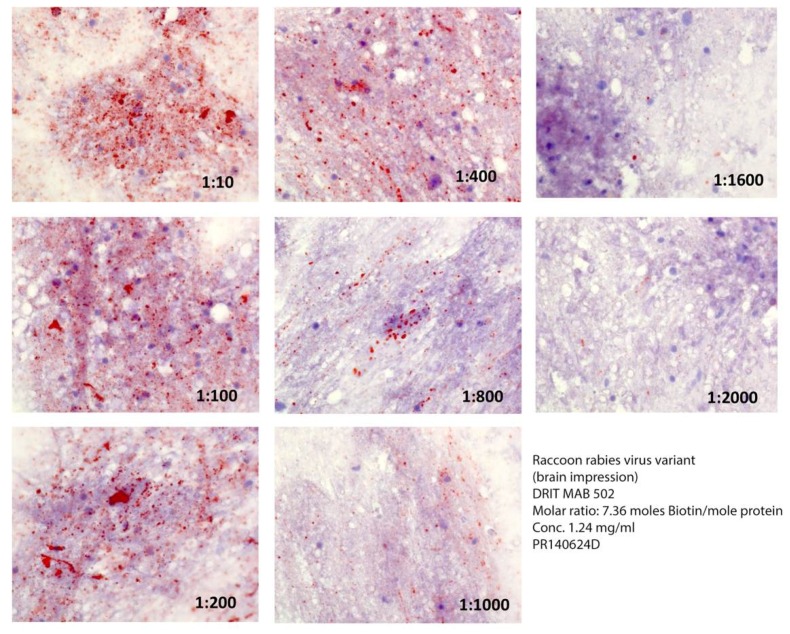
Comparative reactivity of serial dilutions of the DRIT MAB 502 at a ratio of 3.9 moles of biotin per mole of protein against a raccoon rabies virus variant.

The ratio of biotin conjugate to MAB affected the comparative detection of RV inclusions within rabid raccoon brain impressions (Figure 1 and Figure 2) greatly at 7.4 moles, so that RV inclusions were barely detectable at dilutions of or above 1:400 (Figure 1). In contrast, MAB dilution had little effect when the molar ratio was 3.9 moles, even at dilutions in excess of 1/2000 (Figure 2).

**Figure 3:**
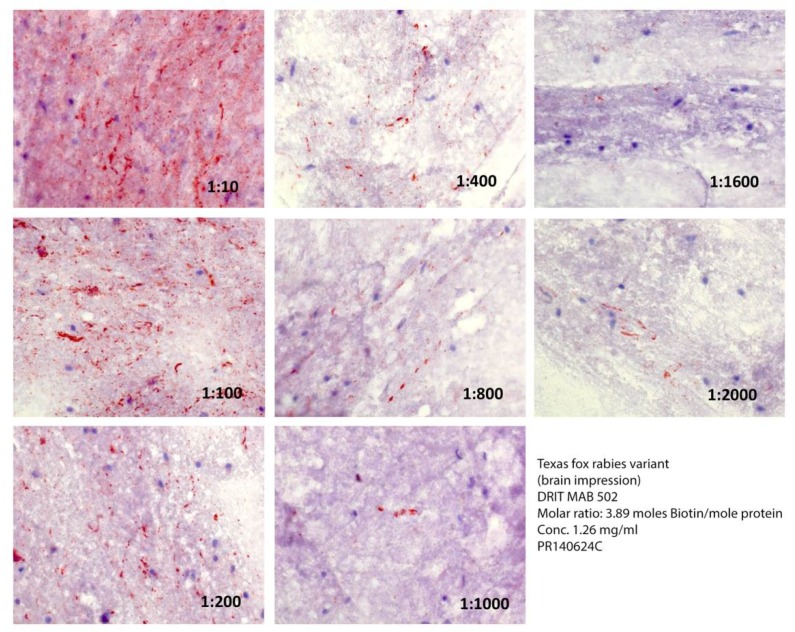
Comparative reactivity of serial dilutions of the DRIT MAB 502 at a ratio of 3.9 moles of biotin per mole of protein against a gray fox rabies virus variant.

Besides biotin conjugation, the choice of RV variant selected to obtain effective working dilutions of the MAB may impact test performance (Figure 3). Although the same relative distribution and abundance of RV inclusions were detected at a MAB dilution of 1/10 in specimens from both an infected raccoon (Figure 2) and a gray fox (Figure 3), the detectability of the latter variant decreased greatly at dilutions of MAB at or above 1/100.

Based upon the biotin and viral variant comparison, 3.9 moles of biotin were chosen for MAB 502 and 802 conjugations. From the 816 animal brain samples examined, the sensitivity and specificity using MABs 502 and 802 were determined to be 98% and 95%, respectively (Table 2). In addition, the positive and negative predictive values were determined to be 89% and 99%, respectively.

The comparison of 27 sample DRIT findings in the PT to the actual FAT results is provided in Table 3. Sensitivity ranged from ~77–100% and specificity ranged from ~86–100%, among the 12–17 participating field laboratories.

## 4. Discussion

As with any rabies diagnostic test, the DRIT includes pre-analytic, analytic and post-analytic stages. Part of the pre-analytic step includes selection of the ABs (MAB or PAB) and their degree of conjugation to biotin. The activity of an AB (e.g., solubility, aggregation, cross-linking, etc.) can be altered by excessive biotinylation [22,23,24]. Typically, a level is targeted below 8–10 biotin molecules per AB. In this investigation, ABs conjugated with four molecules of biotin showed higher sensitivity than ABs conjugated with seven molecules per protein. As this relationship may be affected by variables such as AB isotype, avidity and sequence, the optimal ratio between ABs and biotin molecules within DRIT formulations may differ for other MABs or PABs.

Similarly, the impact of selection for the viral variant in determining the conjugate working dilution cannot be overemphasized, even under the limitation of only two samples in this preliminary study. For example, if a dilution of 1/2,000 was selected for optimal detection of a raccoon RV in the eastern USA and was applied without introspection for the gray fox rabies program, false negative results may have been forthcoming. Such predictions can be extrapolated predictably to the DRIT protocol from decades of use of the FAT, where test sensitivity may be dependent upon viral variant. Significant variation in conjugate concentration and affinity was detected in the USA, particularly for bat RVs (https://www.cdc.gov/rabies/pdf/Low-Affinity-Unavailability-Rabies-Conjugates-NWGRD.pdf). Moreover, this finding was the most parsimonious explanation for the lessened reactivity of the DRIT MAB cocktail towards a mongoose RV variant in South Africa [8]. In that particular case, the working dilution of the MAB conjugate was pre-determined upon the raccoon RV variant. Likely, a higher concentration would have improved detection in that analysis rather than a lack of recognition. The anti-RV nucleocapsid MAB 502 is believed to be a pan-reactor and has detected isolates of all known lyssaviruses [4,17]. Further assessment of comparative reactivity of any DRIT MAB or PAB conjugates should be undertaken against any new lyssaviruses, as well as prospective testing against any unique RV isolates, particularly associated with bats.

In summary of the analytical portion of this investigation, comparative testing of more than 800 animal brain samples generated at reference laboratories in Europe and North America have found a level of sensitivity, specificity, positive and negative predictive value and reproducibility of the DRIT approaching historical data obtained using the FAT under local conditions. Such observations help ensure compatibility for de-centralized rabies diagnosis, as shown recently by enhanced surveillance for raccoon rabies in southern Ontario [5]. Disparate results were obtained by DRIT and FAP for a few samples. In future studies, such samples should be tested by a third method such as nucleic acid-based methods or virus isolation.

As part of quality assurance, laboratories should participate in PT as a part of routine post-analytical considerations [1,2,25]. Widespread use of enhanced wildlife rabies surveillance and application of the DRIT by USDA since 2005 has been critical to accomplish national program goals of rabies prevention and control [19]. To date, more than 90,000 samples have been tested via the DRIT by USDA staff throughout the USA. Improvement of PT scores by USDA personnel in performance of the DRIT over time has been observed, as described here. As additional staff are trained and new field laboratories are established, PT will remain a key aspect for continuing education and reassurance of the reliability of the test at a local level.

Overall, the DRIT, as a presumptive assay for RV diagnosis, is rapid, reproducible and robust under a diversity of testing conditions. Moreover, because the test employs basic light microscopy, the DRIT is less expensive than the routine FAT and could be implemented readily in resource-poor settings, where the majority of human rabies cases occur and laboratory diagnosis is most critical. The fundamental operating principles of the DRIT are based upon objective, sound science and standardized methodology, which beyond basic diagnosis could also be employed for antigenic typing of RV case samples [26]. All of the basic equipment, supplies, and reagents are available commercially and most OIE or WHO rabies reference laboratories have MABs available that will work in the DRIT. Protocols for the production of such MABs are within the public domain, as well as for PAB production. Besides developed countries, investigators have begun to produce conjugates for enhanced surveillance using the DRIT in several developing countries, such as Brazil, China, India and the Philippines, among others [27,28,29,30]. 

Additional commercial availability of any DRIT conjugates (MAB or PAB) would be expected to occur more readily after eventual OIE review, feedback and approval of such methodology, as another suitable and routine test for rabies diagnosis. Clearly, the basic anatomic-pathologic detection of viral inclusions within the CNS and applied utility of the DRIT for routine canine and wildlife rabies surveillance has been demonstrated over the past decade. In addition, extension of test breadth towards additional antigenic and genetic variant detection during enhanced pathogen discovery is anticipated, especially as new lyssavirus species continue to be described [31].

## Figures and Tables

**Table 1:** Comparative testing of brainstem tissues of suspect rabid mammals (N = 816).

Animal	Total Number	Number of Rabid Animals (Positive FAT)
Raccoon	495	105
Skunk (4 taxa)	153	96
Bat (8 taxa)	49	10
Red fox	32	23 *
Dog	25	12 **
Cat	17	1
Coyote	13	0
Cattle	8	7
Gray fox	8	0
Javelina	3	0
Wolf	2	1
Otter	2	0
Marten	2	0
Bobcat	2	0
Deer	1	1
Stone Marten	1	0
Badger	1	0
Woodchuck	1	0
Grey squirrel	1	0

* 5 foxes infected experimentally; ** 5 dogs infected experimentally.

**Table 2:** DRIT mab cocktail diagnostic performance, as compared to the FAT, determined on 816 animal brain samples (Table 1).

	FAT Positive	FAT Negative	Total
DRIT Positive	252	30	282
DRIT Negative	4	530	534
	256	560	
	**Value**	**95% CI**	
Sensitivity	98%	96% to 100%	
Specificity	95%	92% to 96%	
Positive Likelihood Ratio	18	13 to 26	
Negative Likelihood Ratio	0.02	0.01 to 0.04	
Disease prevalence	31%	28% to 35%	
Positive Predictive Value	89%	85% to 93%	
Negative Predictive Value	99%	98% to 100%	

**Table 3:** U.S. National Proficiency Test (PT) Results of DRIT comparison to the FAT. **Samples**: Brain tissue of suspect mammals (n = 27), as described in the history. **Testing**: 2015–17 PT period. **Protocol:** Original DRIT SOP as described and performed by USDA, Wildlife Services staff and 1 state laboratory (12 to 17 participating facilities). **MABs:** Cocktail MAB 502 + MAB 802.

Sample	Animal	History/Signs	FAT Status	DRIT PT Findings	Specificity	Sensitivity
RA-B301	Raccoon	Killed by a vaccinated dog	Negative	1 positive, 14 negative	93%	NA
RA-B312	Dog	Vaccinated; bit member of its owner’s family	Negative	15 negative	100%	NA
RA-B316	Dog	Vaccinated; bit its owner	Negative	1 indeterminate, 14 negative	100%	NA
RA-B319	Cow	Paralysis; unusual vocalizations	Positive	15 positive	NA	100%
RA-B305	Cat	Ataxia; disorientation	Negative	1 positive, 14 negative	93%	
RA-B306	Elk	Farm-raised; neurological signs	Positive	13 positive, 1 indeterminate, 1 negative	NA	93%
RA-B308	Fox	Attacked a dog accompanied by a human	Positive	14 positive, 1 indeterminate	NA	100%
RA-B310	Dog	Unknown vaccination status; bit its owner	Negative	1 positive, 14 negative	93%	NA
RA-B311	Dog	Vaccinated; bit its owner	Negative	1 positive, 14 negative	93%	NA
RA-B300	Cat	Unknown vaccination status; bit its owner	Negative	1 indeterminate, 16 negative	100%	NA
RA-B303	Raccoon	Bit a person	Negative	1 positive, 16 negative	94%	NA
RA-B304	Horse	Incoordination; restlessness; self-mutilation	Positive	14 positive, 3 negative	NA	82%
RA-B315	Goat	Clinical signs for 10 days prior to euthanasia	Positive	10 positive, 2 indeterminate, 3 negative	NA	77%
RA-B317	Dog	Unknown vaccination status; bit a person	Negative	1 indeterminate, 14 negative	100%	NA
RA-B318	Fox	Killed by a vaccinated dog	Negative	14 negative	100%	NA
RA-B322	Dog	Vaccinated; bit its owner	Negative	1 indeterminate, 14 negative	100%	NA
RA-B327	Deer	Apparently blind, with swollen eyes	Positive	15 positive	NA	100%
RA-B328	Dog	Questionable vaccination status; bit a person	Negative	1 indeterminate, 11 negative	100%	NA
RA-B330	Cat	Vaccinated; bit a person	Negative	1 indeterminate, 12 negative	100%	NA
RA-B332	Dog	Unknown vaccination status; bit a person	Negative	1 indeterminate, 12 negative	100%	NA
RA-B334	Sheep	Unvaccinated; compatible signs of encephalitis	Positive	13 positive	NA	100%
RA-B335	Raccoon	Attacked a dog; bit a person	Negative	1 indeterminate, 12 negative	100%	NA
RA-B333	Cat	Questionable vaccination status; bit and scratched a person	Negative	1 positive, 2 indeterminate, 11 negative	92%	NA
RA-B339	Dog	Vaccinated; injured; bit owner	Negative	1 positive, 1 indeterminate, 12 negative	92%	NA
RA-B342	Dog	Unknown vaccination status; chased and bit a person	Negative	1 indeterminate, 13 negative	100%	NA
RA-B347	Dog	Died after a skunk exposure	Negative	2 positive, 12 negative	86%	NA
RA-B349	Cow	Bellowing; hypersalivation; unable to stand	Positive	14 positive	NA	100%
