# Erratum: Rupprecht, C.E., et al. Additional Progress in the Development and Application of a Direct, Rapid Immunohistochemical Test for Rabies Diagnosis. *Vet. Sci.* 2018, *5*, 59

**DOI:** 10.3390/vetsci5030068

**Published:** 2018-07-20

**Authors:** Charles E. Rupprecht, Zhiquan Xiang, Alexandre Servat, Richard Franka, Jordona Kirby, Hildegund C. J. Ertl

**Affiliations:** 1 LYSSA LLC, Atlanta, GA 30333, USA; 2 The Wistar Institute, Philadelphia, PA 19104, USA; jxiang@wistar.org (Z.X.); ertl@wistar.org (H.C.J.E.); 3 OIE/WHO/EU Laboratory for Rabies and Wildlife, French Agency for Food, Environmental and Occupational Health and Safety, 54220 Malzeville, France; Alexandre.SERVAT@anses.fr; 4 CDC, Atlanta, GA 30333, USA; rpf5@cdc.gov; 5 USDA, APHIS, Wildlife Services, Milton, FL 32583, USA; jordona.d.kirby@aphis.usda.gov

Due to an error during production, the order in which Figure 1 and Figure 2 appear and the linking of the Figure 1 and Figure 2 captions in the Results section of the published paper [1] were incorrect. A corrected version of the Figure order and associated captions is provided below. Importantly, these alterations do not modify the primary data, their significance and the related conclusions. The authors apologize for any inconvenience caused to the readers by this minor error. Unfortunately, because of release to the database, the article will not be updated beyond this erratum and the original will remain on the article webpage.

## Figures and Tables

**Figure 1:**
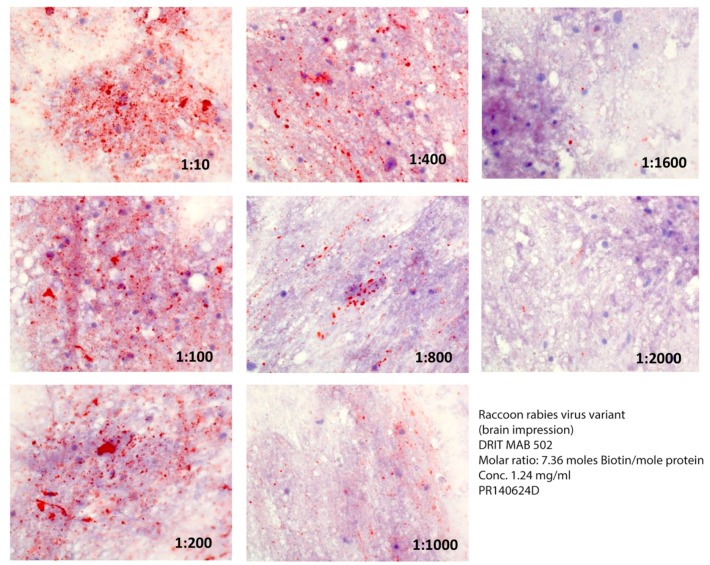
Comparative reactivity of serial dilutions of the DRIT MAB 502 (in PBS) at a ratio of 7.4 moles of biotin per mole of protein against a raccoon rabies virus variant. Rabies virus antigens appear as magenta inclusions against the bluish-purple background of uninfected CNS tissue in Figure 1, Figure 2 and Figure 3.

**Figure 2:**
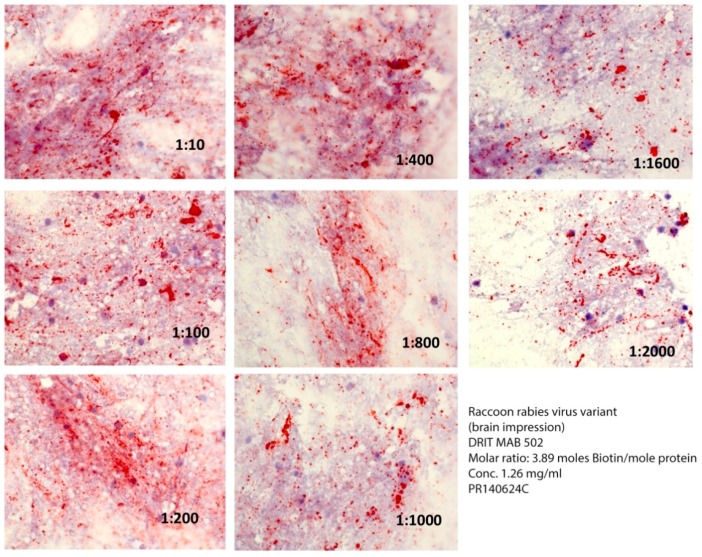
Comparative reactivity of serial dilutions of the DRIT MAB 502 at a ratio of 3.9 moles of biotin per mole of protein against a raccoon rabies virus variant.

## References

[1] Rupprecht C.E., Xiang Z., Servat A., Franka R., Kirby J., Ertl H.C.J. (2018). Additional Progress in the Development and Application of a Direct, Rapid Immunohistochemical Test for Rabies Diagnosis. Vet. Sci..

